# The Association Between Serum FGF21 Level and Coronary Artery Calcification: Impact of the Degree of Insulin Resistance

**DOI:** 10.31083/RCM46781

**Published:** 2026-03-17

**Authors:** Cong Wang, Yingkai Li, Hongyu Peng, Jinghua Liu

**Affiliations:** ^1^Center for Coronary Artery Disease, Beijing Anzhen Hospital, Capital Medical University, 100029 Beijing, China; ^2^Beijing Institute of Heart, Lung, and Blood Vessel Diseases, 100029 Beijing, China

**Keywords:** coronary artery disease, coronary artery calcification, fibroblast growth factor 21, insulin resistance, triglyceride-glucose index

## Abstract

**Background:**

Coronary artery calcification (CAC) is a strong predictor of long-term adverse outcomes in patients with coronary artery disease (CAD). Meanwhile, insulin resistance (IR) is a key metabolic disorder that accelerates CAC progression through multiple pathways. Fibroblast growth factor 21 (FGF21) improves glucolipid metabolism and has been associated with vascular calcification. However, the relationship between serum FGF21 level and CAC severity in patients with varying degrees of IR remains unclear.

**Methods:**

A total of 128 patients with CAD who underwent preprocedural coronary computed tomography angiography and percutaneous coronary intervention were enrolled. Patients were stratified by triglyceride–glucose (TyG) index into high (TyG >8.62, n = 62) and low (TyG ≤8.62, n = 66) groups. Associations between FGF21 levels and severe CAC were analyzed under varying degrees of IR.

**Results:**

In patients with a TyG index >8.62, serum FGF21 levels were significantly lower in those with severe CAC, and were negatively correlated with CAC scores. Multivariable analysis revealed that serum FGF21 levels were independently associated with severe CAC (odds ratio (OR) per 1-standard deviation (SD) increase: 0.261; 95% confidence interval (CI): 0.073, 0.933; *p* < 0.05). In contrast, serum FGF21 levels among patients with a TyG index ≤8.62 did not differ significantly between the severe and non-severe CAC groups, and no independent association between serum FGF21 level and severe CAC was observed after adjustment. Importantly, a significant interaction was observed between the TyG index and FGF21 level (*p* for interaction = 0.035). Moreover, the protective association between FGF21 and CAC was primarily observed in patients with a high TyG index.

**Conclusions:**

Lower serum FGF21 levels in patients with CAD can identify individuals at increased risk of severe CAC, particularly among those with a higher degree of IR. Serum FGF21 levels may serve as a novel biomarker for CAC risk stratification in metabolically susceptible patients.

## 1. Introduction

Coronary artery disease (CAD) remains the major cause of mortality globally, with its pathogenesis intricately linked to atherosclerosis [1]. A critical hallmark of advanced atherosclerosis is the development of coronary artery calcification (CAC), which is recognized as a notable independent predictor associated with future cardiovascular events and all-cause mortality [2]. CAC is therefore a key target in the prevention and treatment of CAD, especially in CAD patients with multivessel disease [3, 4]. The progression of CAC is accelerated in individuals with metabolic disorders, particularly those with insulin resistance (IR) [5, 6]. IR fosters a pro-atherogenic milieu through multiple pathways, including chronic inflammation, endothelial dysfunction, dyslipidemia, and oxidative stress [7]. Collectively, these factors collectively promote the transition of vascular smooth muscle cells (VSMCs) toward an osteoblast-like phenotype, driving the deposition of hydroxyapatite crystals in the coronary arteries, which is a typical pathophysiological feature of CAC [8]. The triglyceride-glucose (TyG) index is a practical and reliable surrogate marker of IR that can independently predict both the presence and progression of CAC [9, 10]. Despite its established clinical significance, the precise molecular mechanisms linking IR to the accelerated calcification process have not been fully elucidated, highlighting the need for novel biomarkers and pathophysiological insights. Fibroblast growth factor 21 (FGF21), a member of the fibroblast growth factor family, is recognized as a pivotal metabolic regulator with multifaceted roles in glucose and lipid homeostasis [11]. FGF21 is primarily secreted by the liver and acts by enhancing insulin sensitivity, promoting glucose uptake, and improving lipid profiles [12]. These properties make FGF21 a critical defender against metabolic disorders such as IR, type 2 diabetes, and obesity [13, 14]. Our previous studies showed that FGF21 attenuated vascular calcification both *in vivo* and *in vitro* by inhibiting endoplasmic reticulum stress and reducing oxidative stress [15, 16, 17]. Hence, the exogenous administration of FGF21 could be a promising therapeutic intervention for vascular calcification [18].

The aim of this study was therefore to evaluate the association between serum FGF21 levels and the severity of CAC in a well-characterized cohort of patients with varying degrees of IR. By elucidating this relationship, we seek to identify a potential biomarker for risk stratification in metabolically susceptible patients with CAC.

## 2. Materials and Methods

### 2.1 Study Population

This retrospective observational study consecutively included 128 CAD patients aged ≥18 years treated at Beijing Anzhen Hospital between December 2020 and December 2022. Patients underwent coronary computed tomography angiography (CCTA) and CAC scoring prior to coronary angiography (CAG) and indicated percutaneous coronary intervention (PCI). CAD was defined as the presence of at least one major coronary vessel (left main, left anterior descending, left circumflex, or right coronary artery) with ≥50% diameter stenosis, as assessed by CAG [19]. The exclusion criteria were as follows: missing data for fasting blood glucose (FBG) or triglyceride levels; suspected familial hypertriglyceridemia (triglyceride ≥5.65 mmol/L); severe hepatic or renal dysfunction; severe heart failure or cardiogenic shock (left ventricular ejection fraction <35%); history of previous coronary intervention or coronary artery bypass graft, infectious disease, or malignant tumor.

### 2.2 Clinical Data Collection 

General patients’ data were extracted from the hospital’s electronic medical record system, including demographic and clinical characteristics, laboratory test results, angiographic results, and procedural details. Blood samples were collected after overnight fasting. Routine biochemical parameters, including FBG and lipid measurements, were processed the same day in the central laboratory according to standardized laboratory techniques. The TyG index was calculated using the formula: Ln [triglyceride (mg/dL) × FBG (mg/dL)/2] [20].

### 2.3 CAC Score 

CCTA scans were conducted using a 256-detector row CT system (Revolution CT, GE Healthcare, Milwaukee, WI, USA). Non-contrast cardiac CT imaging was obtained prior to CCTA, and all procedures adhered to the standards outlined in the Society of Cardiovascular Computed Tomography guidelines [21]. The CAC score was calculated automatically using the Agatston method [22], independent of any clinical information. Patients with a score ≥400 were considered to have severe CAC [23].

### 2.4 Measurement of FGF21

After admission, fasting venous blood samples (5 mL) were collected from all patients in the morning using serum separator tubes. The samples were allowed to clot for 30 minutes at room temperature before centrifugation at 3000 r/min for 10 minutes. Subsequently, the serum was aliquoted into 1.5 mL EP tubes and stored at –80 °C. Serum FGF21 was measured by Enzyme-Linked Immunosorbent Assay (DF2100, R&D Systems, Minneapolis, MN, USA), according to the manufacturer’s instructions.

### 2.5 Statistical Analysis 

The Kolmogorov-Smirnov test was applied to assess the normality of continuous variables. Data were expressed as the mean ± standard deviation (SD) or median (interquartile range, IQR). Comparisons were performed using Student’s *t* test or the Mann-Whitney *U* test as appropriate. Categorical variables were presented as numbers (percentages) and compared using the chi-square test. Correlations between FGF21 levels and CAC score were evaluated using Spearman’s tests.

Logistic regression models were constructed to evaluate the association between FGF21 and severe CAC in the overall population and subgroups with different TyG index levels. Odds ratios (ORs) were calculated per one SD increase in serum FGF21 levels. Covariates considered clinically relevant to severe CAC were selected in the multivariable analyses. The fully adjusted model was adjusted for sex, age, body mass index (BMI), history of hypertension, diabetes, dyslipidemia, smoking, and the use of antidiabetic agents. Multiplicative interaction terms were included in the adjusted models to assess whether TyG index levels modify the association between FGF21 and severe CAC. Receiver operating characteristic (ROC) curves were constructed to assess the diagnostic performance of serum FGF21 in predicting severe CAC. Youden’s index was calculated, together with the maximum value which corresponds to the optimal cutoff value of FGF21 levels. Sensitivity analyses were performed to assess the robustness of the primary findings. First, serum FGF21 levels were converted into a categorical variable based on the optimal cutoff identified by Youden’s index, and logistic regression analyses were re-performed to assess the robustness of the associations. Second, restricted cubic spline (RCS) curves (3 knots) were applied to explore the dose–response association between serum FGF21 levels and severe CAC in the overall population as well as in the subgroups stratified by TyG index.

Statistical analyses were performed with SPSS 26.0 (IBM SPSS, Armonk, NY, USA) and R software (version 4.4.3, R Foundation for Statistical Computing, Vienna, Austria). A two-sided *p*-value < 0.05 was considered statistically significant.

## 3. Results

### 3.1 Clinical Characteristics of the Study Population

**Fig. 1.**
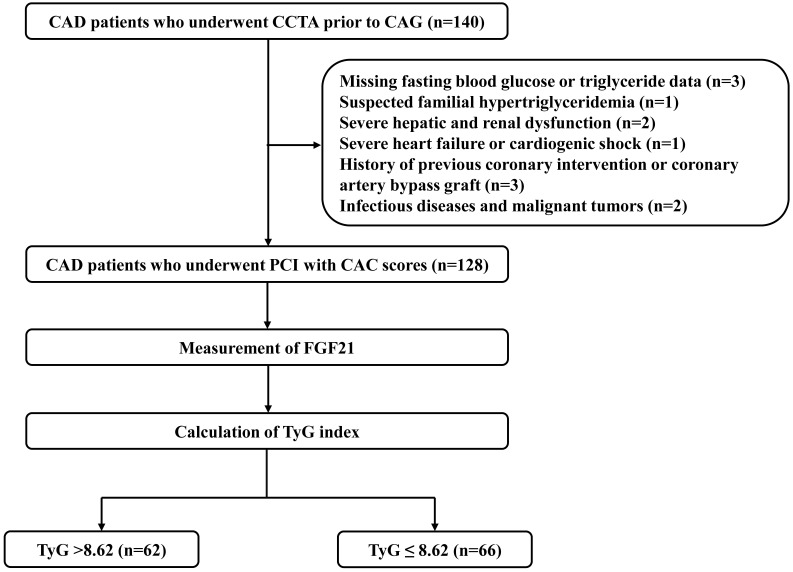
**Flow diagram for patient enrollment**. Abbreviations: CAC, coronary artery calcification; CAD, coronary artery disease; CAG, coronary angiography; CCTA, coronary computed tomography angiography; FGF21, fibroblast growth factor 21; PCI, percutaneous coronary intervention; TyG, triglyceride-glucose.

A total of 128 CAD patients were enrolled and divided into high TyG index (n = 62, TyG index >8.62) and low TyG index groups (n = 66, TyG index ≤8.62) based on the median value [8.62 (8.41–9.01)] (Fig. 1). The baseline characteristics of CAD patients with varying degrees of IR are presented in Table 1. Compared to patients with lower TyG index, those with a TyG index >8.62 had higher BMI, a higher incidence of diabetes and dyslipidemia, significantly higher levels of FBG, total cholesterol, and triglycerides, and lower levels of high-density lipoprotein cholesterol (all *p* < 0.05). The distribution of the TyG index in severe and non-severe CAC groups is presented in **Supplementary Fig. 1**.

**Table 1.** **Baseline characteristics of CAD patients with varying degrees of IR**.

Variables			Overall (n = 128)	TyG index >8.62 (n = 62)	TyG index ≤8.62 (n = 66)	*p-*value
Demographics						
	Age (years)		58 ± 10	57 ± 9	59 ± 11	0.186
	Male		108 (84.4)	52 (83.9)	56 (84.8)	0.879
	BMI (kg/m^2^)		26.9 ± 3.3	27.6 ± 3.4	26.3 ± 3.2	0.023
	Smoking		67 (52.3)	35 (56.5)	32 (48.5)	0.367
Medical history						
	Hypertension		83 (64.8)	42 (67.7)	41 (62.1)	0.506
	Diabetes		45 (35.2)	30 (48.4)	15 (22.7)	0.002
	Dyslipidemia		47 (36.7)	44 (71.0)	3 (4.5)	<0.001
	Prior MI		33 (25.8)	16 (25.8)	17 (25.8)	0.995
	Prior stroke		12 (9.4)	6 (9.7)	6 (9.1)	0.909
	Heart failure		9 (7.0)	5 (8.1)	4 (6.1)	0.658
Laboratory tests						
	Creatinine (µmol/L)		73.2 (64.1–83.0)	74.7 (66.6–85.7)	70.3 (62.0–82.6)	0.234
	eGFR (mL/min/1.73 m^2^)		96.8 (86.8–105.1)	95.1 (86.1–103.8)	96.8 (87.9–106.5)	0.414
	FBG (mmol/L)		5.1 (4.5–6.1)	5.8 (5.0–6.6)	5.2 (4.7–6.2)	<0.001
	TC (mmol/L)		3.6 (3.1–4.1)	3.8 (3.3–4.6)	3.4 (3.1–3.8)	0.006
	TG (mmol/L)		1.4 (1.1–2.0)	2.0 (1.6–2.3)	1.1 (0.9–1.3)	<0.001
	LDL-C (mmol/L)		1.9 (1.5–2.3)	2.0 (1.5–2.7)	1.8 (1.5–2.2)	0.154
	HDL-C (mmol/L)		0.95 (0.82–1.1)	0.9 (0.8–1.0)	1.0 (0.9–1.2)	0.001
	TyG index		8.62 (8.41–9.01)	9.01 (8.86–9.34)	8.41 (8.13–8.54)	<0.001
	Serum FGF21 (pg/mL)		257.6 (156.5–370.9)	270.6 (163.7–452.2)	251.7 (139.4–337.3)	0.291
CAG and PCI results						
	CAC score		310.0 (97.6–548.5)	294.3 (96.4–600.0)	340.9 (96.8–547.1)	0.830
	Severe CAC		48 (37.5)	23 (37.1)	25 (37.9)	0.927
	Multivessel disease		95 (74.2)	45 (72.6)	50 (75.8)	0.569
	Target vessel territory					
		LAD	50 (39.1)	25 (40.3)	25 (37.9)	0.702
		LCX	11 (8.6)	4 (6.5)	7 (10.6)	
		RCA	67 (52.3)	33 (53.2)	34 (51.5)	
	Number of stents					
		0	34 (26.6)	16 (25.8)	18 (27.3)	0.710
		1	28 (21.9)	12 (19.4)	16 (24.2)	
		2	39 (30.5)	22 (35.5)	17 (25.8)	
		≥3	27 (21.1)	12 (19.3)	15 (22.7)	
Medications during hospitalization						
	Aspirin		128 (100.0)	62 (100.0)	66 (100.0)	-
	P_2_Y_12_ inhibitors		128 (100.0)	62 (100.0)	66 (100.0)	-
	Statins		128 (100.0)	62 (100.0)	66 (100.0)	-
	Antidiabetic agents		40 (31.3)	25 (40.3)	15 (22.7)	0.032
		Oral hypoglycemic agents	10 (7.8)	6 (9.7)	4 (6.1)	0.446
		Insulin	35 (27.3)	21 (33.9)	14 (21.2)	0.108

Data are presented as the mean ± SD, median (IQR), or n (%). Abbreviations: BMI, body mass index; CAC, coronary artery calcification; CAD, coronary artery disease; CAG, coronary angiography; eGFR, estimated glomerular filtration rate; FBG, fasting blood glucose; FGF21, fibroblast growth factor 21; HDL-C, high-density lipoprotein cholesterol; IR, insulin resistance; LAD, left anterior descending artery; LCX, left circumflex artery; LDL-C, low-density lipoprotein cholesterol; MI, myocardial infarction; PCI, percutaneous coronary intervention; RCA, right coronary artery; TC, total cholesterol; TG, triglyceride; TyG, triglyceride-glucose.

### 3.2 Association of Serum FGF21 Levels With CAC After Stratification for the Degree of IR

**Fig. 2.**
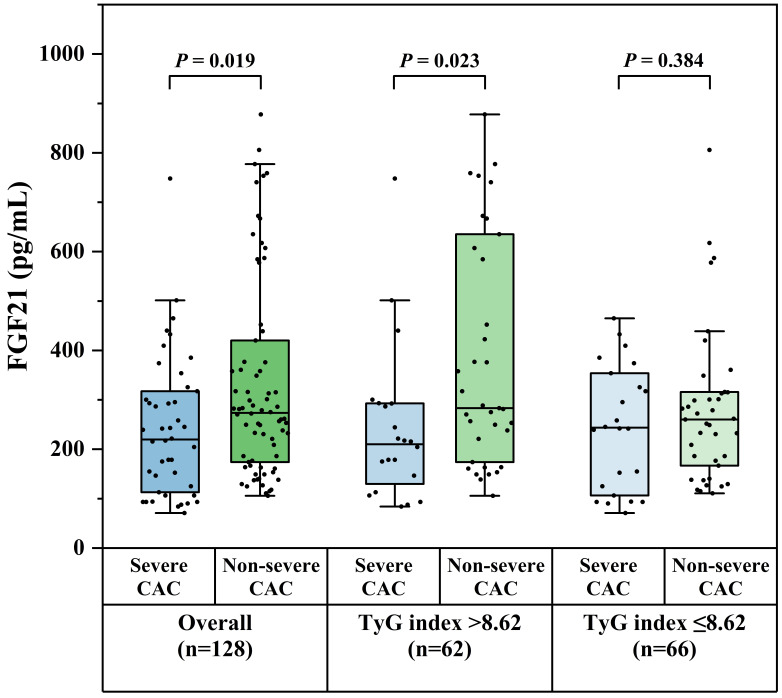
**Comparison of serum FGF21 levels between severe and non-severe CAC groups in CAD patients with varying degrees of IR**. Abbreviations: CAC, coronary artery calcification; CAD, coronary artery disease; FGF21, fibroblast growth factor 21; IR, insulin resistance; TyG, triglyceride-glucose.

Table 1 shows the comparison of serum FGF21 levels and CAC scores in CAD patients with varying degrees of IR. No significant differences in FGF21 levels and CAC scores were observed between the high and low TyG index groups. In the overall population, serum FGF21 levels were significantly lower in patients with severe CAC compared to those with non-severe CAC [219.7 (111.5–319.6) vs. 273.7 (172.2–424.8) pg/mL, *p* = 0.019]. When stratified by TyG index, this difference remained significant in patients with a TyG index >8.62, among whom serum FGF21 levels were significantly lower in the severe CAC group [210.0 (121.5–293.1) vs. 283.2 (174.0–635.3) pg/mL, *p* = 0.023]. In contrast, no significant difference in FGF21 level was observed between severe and non-severe CAC groups in patients with a TyG index ≤8.62 (**Supplementary Table 1**, Fig. 2). Correlation analyses revealed that serum FGF21 levels were negatively correlated with CAC score in the overall population (*r* = –0.203, *p* = 0.029) and in the subgroup with TyG index >8.62 (*r* = –0.275, *p* = 0.042). However, no significant correlation was found between FGF21 levels and the CAC score in patients with a TyG index ≤8.62 (Table 2, **Supplementary Fig. 2**).

**Table 2.** **Correlations between serum FGF21 level and CAC score in CAD patients with varying degrees of IR**.

Variable	Overall (n = 128)		TyG index >8.62 (n = 62)		TyG index ≤8.62 (n = 66)	
	*r* with CAC score	*p*-value	*r* with CAC score	*p*-value	*r* with CAC score	*p*-value
Serum FGF21 (pg/mL)	–0.203	0.029	–0.275	0.042	–0.133	0.309

Abbreviations: CAC, coronary artery calcification; CAD, coronary artery disease; FGF21, fibroblast growth factor 21; IR, insulin resistance; TyG, triglyceride-glucose.

### 3.3 Predictive and Diagnostic Value of Serum FGF21 for Severe CAC

**Fig. 3.**
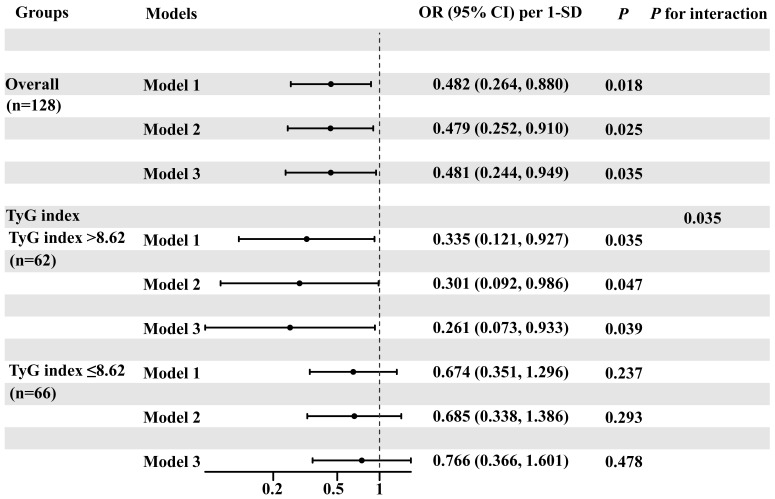
**Forest plot of logistic regression analyses for the association between serum FGF21 and severe CAC in CAD patients with varying degrees of IR**. Abbreviations: CAC, coronary artery calcification; CAD, coronary artery disease; CI, confidence interval; FGF21, fibroblast growth factor 21; IR, insulin resistance; OR, odds ratio; SD, standard deviation; TyG, triglyceride-glucose.

Table 3 shows the results of logistic regression analyses. In the overall population, serum FGF21 levels were independently associated with severe CAC after full adjustment. The OR per 1-SD increase in FGF21 level was 0.481 (95% confidence interval [CI]: 0.244, 0.949; *p* < 0.05). Similarly, in the subgroup with TyG index >8.62, elevated FGF21 level remained an independent protective factor against severe CAC after multivariate adjustment (OR [95% CI]: 0.261 [0.073, 0.933], *p* = 0.039). In contrast, no significant association was found between the serum FGF21 level and severe CAC in patients with a lower TyG index (Table 3). Moreover, a significant interaction was observed between the TyG index and serum FGF21 level in predicting severe CAC (*p* for interaction = 0.035, Fig. 3).

**Table 3.** **Univariate and multivariate logistic regression analyses of severe CAC in CAD patients with varying degrees of IR**.

FGF21 (per 1-SD)	Overall (n = 128)		TyG index >8.62 (n = 62)		TyG index ≤8.62 (n = 66)	
	OR (95% CI)	*p*-value	OR (95% CI)	*p*-value	OR (95% CI)	*p*-value
Model 1	0.482 (0.264, 0.880)	0.018	0.335 (0.121, 0.927)	0.035	0.674 (0.351, 1.296)	0.237
Model 2	0.479 (0.252, 0.910)	0.025	0.301 (0.092, 0.986)	0.047	0.685 (0.338, 1.386)	0.293
Model 3	0.481 (0.244, 0.949)	0.035	0.261 (0.073, 0.933)	0.039	0.766 (0.366, 1.601)	0.478

Abbreviations: CAC, coronary artery calcification; CAD, coronary artery disease; CI, confidence interval; FGF21, fibroblast growth factor 21; IR, insulin resistance; OR, odds ratio; SD, standard deviation; TyG, triglyceride-glucose. Model 1: unadjusted. Model 2: adjusted for sex, age, and BMI. Model 3: adjusted for Model 2 covariates plus hypertension, diabetes, dyslipidemia, smoking, and use of antidiabetic agents.

**Supplementary Fig. 3** shows the diagnostic performance of FGF21 for severe CAC, as determined by ROC curve analysis. In the overall population, the area under the curve (AUC) for serum FGF21 was 0.632 (95% CI: 0.525, 0.739; *p* = 0.019). The optimal cut-off value was determined as 114.2 pg/mL, with a sensitivity of 97.3% and specificity of 26.2%. For patients with a TyG index >8.62, the serum FGF21 level also showed significant predictive value for severe CAC, with an AUC of 0.686 (95% CI: 0.541, 0.831; *p* = 0.023). The optimal cut-off value for the high TyG index subgroup was 229.9 pg/mL, with a sensitivity of 71.4% and specificity of 65.0% (**Supplementary Table 2**).

### 3.4 Sensitivity Analyses

Consistent with the main findings, when FGF21 was converted into a categorical variable, elevated FGF21 levels (i.e., greater than the optimal cutoff) remained significantly associated with a lower risk of severe CAC in both the overall population (OR [95% CI]: 0.206 [0.075, 0.570], *p* = 0.002) and in the high TyG index subgroup (OR [95% CI]: 0.113 [0.024, 0.540], *p* = 0.006) after full adjustment. In contrast, no significant association was observed in the low TyG index subgroup (**Supplementary Table 3**). Furthermore, RCS curves revealed a generally linear, inverse dose-response relationship between serum FGF21 levels and the risk of severe CAC in both the overall population and the high TyG subgroup (*p*-overall = 0.057 and 0.074, respectively; both *p*-nonlinear > 0.05). In contrast, no significant dose–response association was detected among patients with lower TyG index (**Supplementary Fig. 4**).

## 4. Discussion

The present study found that the association between serum FGF21 level and severe CAC in a cohort of patients with CAD was significantly modified by the degree of IR, as assessed by the TyG index. Specifically, among patients with a higher degree of IR (TyG index >8.62), the serum FGF21 level was significantly lower in those with severe CAC, was negatively correlated with the CAC score, and was identified as an independent protective factor against severe CAC. In contrast, no significant association was observed between FGF21 and CAC in patients with a lower degree of IR (TyG index ≤8.62). Importantly, a significant interaction was found between the TyG index and FGF21 levels in predicting severe CAC. To our knowledge, this is the first study to elucidate the impact of the degree of IR on the association between serum FGF21 and severe CAC in CAD patients. IR, characterized by reduced insulin sensitivity and responsiveness, is a hallmark of type 2 diabetes and is strongly linked to cardiovascular diseases [24, 25]. The TyG index is a convenient and reliable alternative indicator for IR that is significantly correlated with the hyperinsulinemic-euglycemic clamp and is widely used in clinical practice and research [20, 26, 27]. In the current study, CAD patients with a higher TyG index had a higher prevalence of diabetes and elevated FBG levels, further supporting its clinical utility as a marker reflecting the severity of IR. Meanwhile, given the close relationship between IR and glucose-lipid metabolism, FGF21 has garnered attention as a key metabolic regulator involved in this process [13, 28, 29]. In addition to improving glucolipid metabolism and reducing inflammation, FGF21 was also shown in our earlier studies to alleviate vascular calcification by reducing endoplasmic reticulum stress and oxidative stress. However, the association between serum FGF21 levels and CAC, as well as the specific pathological mechanisms involved, remains unclear. In this study, we found that FGF21 levels in CAD patients were significantly lower in the severe CAC group. In addition, they were negatively correlated with the degree of calcification, suggesting that higher FGF21 levels may reflect a compensatory metabolic response associated with lower calcification severity.

Notably, this relationship was significantly modified by the degree of IR, our findings revealed a significant interaction between the TyG index and FGF21 levels in predicting severe CAC, with the protective association of FGF21 primarily observed in patients with a higher degree of IR (TyG index >8.62). Regarding the likely pathological mechanism, IR leads to impaired glycemic stability and exposes patients to a persistent state of metabolic stress characterized by dysregulated glucose and lipid metabolism, chronic inflammation, and oxidative stress [24, 30]. These pathological processes not only exacerbate vascular injury and endothelial dysfunction, but also promote the osteogenic trans-differentiation of VSMCs, thereby driving the development and progression of CAC [7, 31, 32]. In this abnormal metabolic environment, the liver and other tissues increase their secretion of FGF21 as a compensatory protective response to restore metabolic homeostasis and mitigate metabolic damage [33, 34]. Therefore, in individuals with a higher degree of IR, elevated serum FGF21 levels may reflect a more effective adaptive response. Patients capable of mounting a stronger FGF21 response can attenuate atherosclerosis and vascular calcification through multiple mechanisms, such as improving insulin sensitivity, reducing cardiac lipotoxicity, suppressing inflammatory pathways, and alleviating oxidative stress [35, 36]. This may partially explain the significant negative correlation we observed between the FGF21 level and the severity of CAC, as well as its independent predictive value for severe CAC. On the other hand, in individuals with relatively healthy metabolism (i.e., those with a lower TyG index), baseline FGF21 levels may be sufficient to maintain vascular homeostasis, meaning that any fluctuations in the level are less critical for regulating the calcification process. Furthermore, given that the low TyG index subgroup typically exhibits a lower CAC burden, the compensatory upregulation of FGF21 tends to be weaker, resulting in further attenuation of the association between serum FGF21 levels and CAC. Taken together, the observed negative correlation in the overall population may reflect the average effect of FGF21 on severe CAC under the heterogeneous metabolic states of CAD patients. The significant interaction between TyG and FGF21 indicates that the observed protective association of FGF21 is primarily driven by individuals with a higher TyG index.

Although IR is known to be closely related to vascular calcification and FGF21 secretion, our study did not find significant differences in CAC scores or serum FGF21 levels between the high and low TyG index groups. There may be several reasons for this. First, CAC is influenced by multiple factors, including age, gender, ethnicity, and cumulative exposure to other traditional cardiovascular risk factors, and is not entirely attributable to IR [37]. Therefore, the degree of IR defined by the TyG index may not directly translate into group-level differences in the calcification burden within this relatively small CAD cohort. Second, FGF21 is a stress-induced cytokine produced primarily by the liver, adipose tissue, and skeletal muscle. Consequently, circulating FGF21 levels are regulated by various factors beyond IR, including liver function, inflammation, and drug treatments [38, 39]. These factors may partially attenuate any significant differences between groups. Third, the relatively small sample size may have limited the ability to identify modest group-level differences.

In summary, our findings indicate the association of FGF21 with CAC is not static, but is modulated by the underlying metabolic environment. The interaction between the degree of IR and FGF21 is crucial for predicting CAC risk, and the inverse association of FGF21 with CAC is more pronounced in CAD patients with impaired metabolism. Therefore, in individuals with IR, FGF21 may not only serve as a valuable biomarker for CAC risk, but also as a complementary indicator for risk stratification in metabolically susceptible CAD patients. Given the growing evidence supporting FGF21 as a potential therapeutic target for various metabolic disorders [40, 41, 42], regular monitoring of FGF21 levels and the implementation of early preventive measures in high-risk populations may hold significant clinical importance.

### Limitations

The present study has several limitations that should be considered. First, as a single-center, observational study, our analysis cannot establish causality between FGF21 and CAC, and the influence of selection bias or unmeasured confounders cannot be fully ruled out. Furthermore, the relatively small sample size limits the statistical power of the analyses. This potentially affects the stability of our estimates, particularly in the subgroup analyses, and limits the external validity of our findings. Moreover, the TyG cutoff used in this study was derived from the median value of our cohort, rather than an externally validated threshold, and thus may not be directly applicable to other populations. Therefore, the current findings should be interpreted with caution, and larger studies are needed to validate the observed associations. Second, the TyG index was measured only at baseline and did not capture potential fluctuations over time, which may lead to misclassification bias. Finally, the study population was derived from a single-center East Asian cohort, and hence our findings may not be generalizable to other ethnic groups. Future prospective studies with larger sample sizes and multi-time point metabolic assessments are needed to further elucidate the complex relationship between FGF21, IR, and CAC.

## 5. Conclusions

This study found that the association between FGF21 and CAC was significantly modified by the degree of IR. In CAD patients with a higher degree of IR (TyG index >8.62), the serum FGF21 level was negatively correlated with CAC severity and served as an independent predictor associated with a lower risk of severe CAC. These findings suggest that FGF21 may be used as a novel biomarker to identify metabolically susceptible individuals at increased risk of CAC, with potential value for risk stratification and early prevention.

## Data Availability

The data regarding this article will be shared by the corresponding author upon reasonable request.

## Abbreviations

AUC, area under the curve; BMI, body mass index; CAC, coronary artery calcification; CAD, coronary artery disease; CAG, coronary angiography; CCTA, coronary computed tomography angiography; CI, confidence interval; eGFR, estimated glomerular filtration rate; FBG, fasting blood glucose; FGF21, fibroblast growth factor 21; HDL-C, high-density lipoprotein cholesterol; IQR, interquartile range; IR, insulin resistance; LAD, left anterior descending artery; LCX, left circumflex artery; LDL-C, low-density lipoprotein cholesterol; MI, myocardial infarction; OR, odds ratio; PCI, percutaneous coronary intervention; RCA, right coronary artery; ROC, receiver operating characteristic; SD, standard deviation; TC, total cholesterol; TG, triglyceride; TyG, triglyceride-glucose; VSMCs, vascular smooth muscle cells.

## Supplementary Material

Supplementary material associated with this article can be found, in the online version, at https://doi.org/10.31083/RCM46781.
