# Supplementary material for: The Association Between Serum FGF21 Level and Coronary Artery Calcification: Impact of the Degree of Insulin Resistance
Source: Rev Cardiovasc Med. 2026 Mar 17;27(3):46781. doi: 10.31083/RCM46781 (PMC13036554; doi:10.31083/RCM46781)

**Supplementary material**

Supplementary Table 1. Comparison of serum FGF21 levels between severe and non-severe CAC groups in CAD patients with varying degrees of IR

| Variables | Overall  (n=128) | | | TyG index >8.62  (n=62) | | | TyG index ≤8.62  (n=66) | | |
| --- | --- | --- | --- | --- | --- | --- | --- | --- | --- |
|  | Severe CAC  (n=48) | Non-severe CAC  (n=80) | *P-*value | Severe CAC  (n=23) | Non-severe CAC  (n=39) | *P-*value | Severe CAC  (n=25) | Non-severe CAC  (n=41) | *P-*value |
| Serum FGF21 (pg/mL) | 219.7 (111.5-319.6) | 273.7 (172.2-424.8) | 0.019 | 210.0 (121.5-293.1) | 283.2 (174.0-635.3) | 0.023 | 243.6 (103.3-358.9) | 260.2 (166.9-315.8) | 0.384 |

Abbreviations: CAC, coronary artery calcification; CAD, coronary artery disease; FGF21, fibroblast growth factor 21; IR, insulin resistance; TyG, triglyceride-glucose index.

Supplementary Table 2. ROC curve analyses of serum FGF21 for diagnosing severe CAC in CAD patients with varying degrees of IR

| **For severe CAC** | AUC (95% CI) | *P-*value | Se (%) | Sp (%) | Cut-off |
| --- | --- | --- | --- | --- | --- |
| **Overall (n=128)** |  |  |  |  |  |
| FGF21 (pg/mL) | 0.632 (0.525,0.739) | 0.019 | 97.3 | 26.2 | 114.2 |
| **TyG index >8.62 (n=62)** |  |  |  |  |  |
| FGF21 (pg/mL) | 0.686 (0.541, 0.831) | 0.023 | 71.4 | 65.0 | 229.9 |
| **TyG index ≤8.62 (n=66)** |  |  |  |  |  |
| FGF21 (pg/mL) | 0.568 (0.407, 0.728) | 0.384 | 97.4 | 27.3 | 113.0 |

Abbreviations: AUC, area under the curve; CAC, coronary artery calcification; CAD, coronary artery disease; CI: confidence interval; FGF21, fibroblast growth factor 21; IR, insulin resistance; ROC, receiver operating characteristic; Se, sensitivity; Sp, specificity; TyG, triglyceride-glucose index.

Supplementary Table 3. Univariate and multivariate logistic regression analyses of severe CAC using categorical FGF21 based on the optimal cutoff value

| Elevated FGF21 levels | Overall  (n=128) | | TyG index >8.62  (n=62) | | TyG index ≤8.62  (n=66) | |
| --- | --- | --- | --- | --- | --- | --- |
|  | FGF21 >114.2 pg/mL | | FGF21 >229.9 pg/mL | | FGF21 >113.0 pg/mL | |
|  | OR (95% CI) | *P-*value | OR (95% CI) | *P-*value | OR (95% CI) | *P-*value |
| Model 1 | 0.203 (0.079, 0.519) | 0.001 | 0.245 (0.081, 0.738) | 0.012 | 0.070 (0.008, 0.631) | 0.018 |
| Model 2 | 0.190 (0.072, 0.500) | 0.001 | 0.124 (0.030, 0.523) | 0.004 | 0.088 (0.009, 0.824) | 0.033 |
| Model 3 | 0.206 (0.075, 0.570) | 0.002 | 0.113 (0.024, 0.540) | 0.006 | 0.126 (0.010, 1.553) | 0.106 |

Abbreviations: CAC, coronary artery calcification; CI: confidence interval; FGF21, fibroblast growth factor 21; OR: odds ratio; TyG, triglyceride-glucose index.

Model 1: unadjusted. Model 2: adjusted for sex, age, and BMI. Model 3: adjusted for Model 2 covariates plus hypertension, diabetes, dyslipidemia, smoking, and use of antidiabetic agents.

Supplementary Fig. 1 The distribution of TyG index in severe and non-severe CAC groups


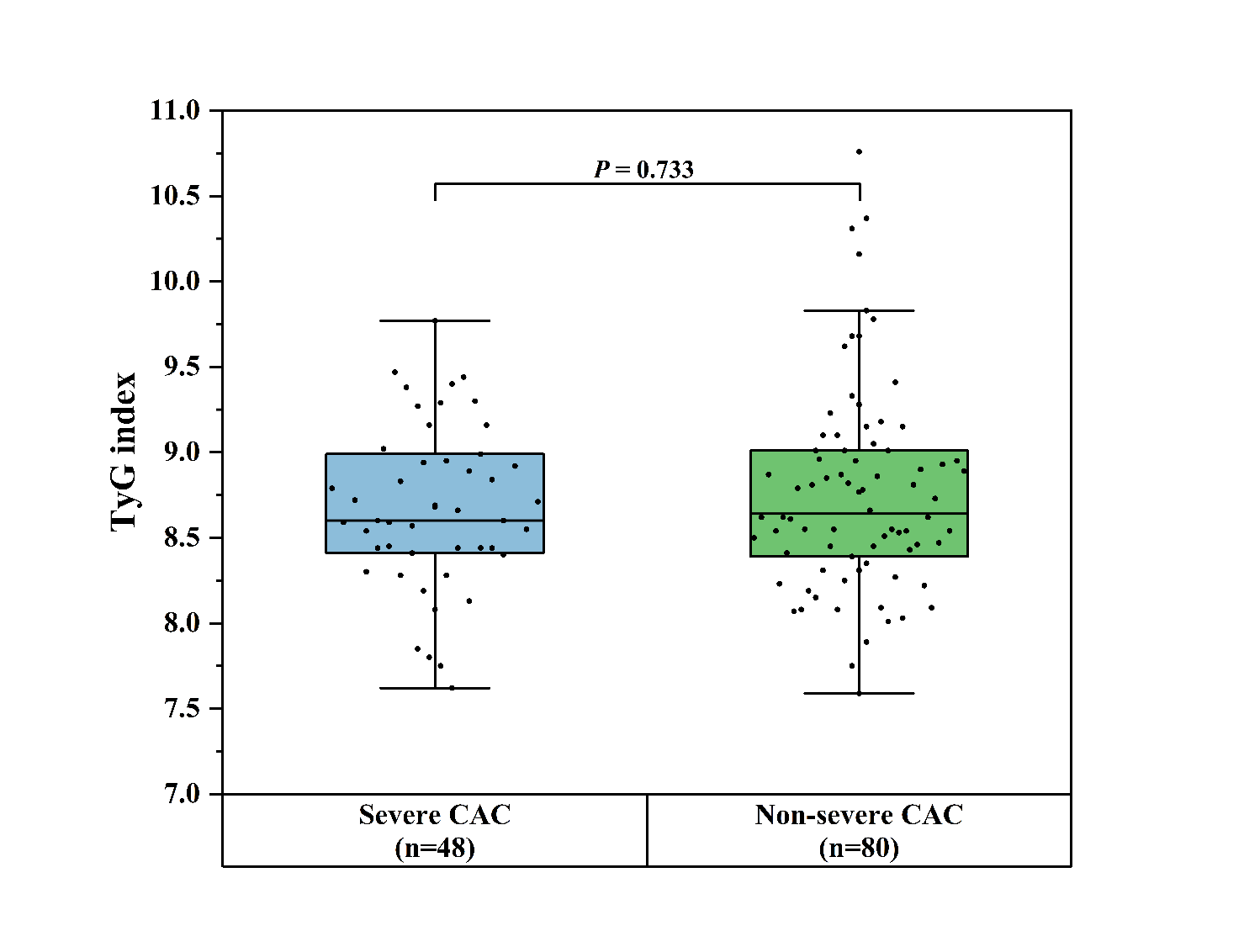
Abbreviations: CAC, coronary artery calcification; TyG, triglyceride-glucose index.

Supplementary Fig. 2 Correlation between FGF21 levels and CAC scores in CAD patients with varying degrees of IR

Abbreviations: CAC, coronary artery calcification; CAD, coronary artery disease; FGF21, fibroblast growth factor 21; IR, insulin resistance; TyG, triglyceride-glucose index.


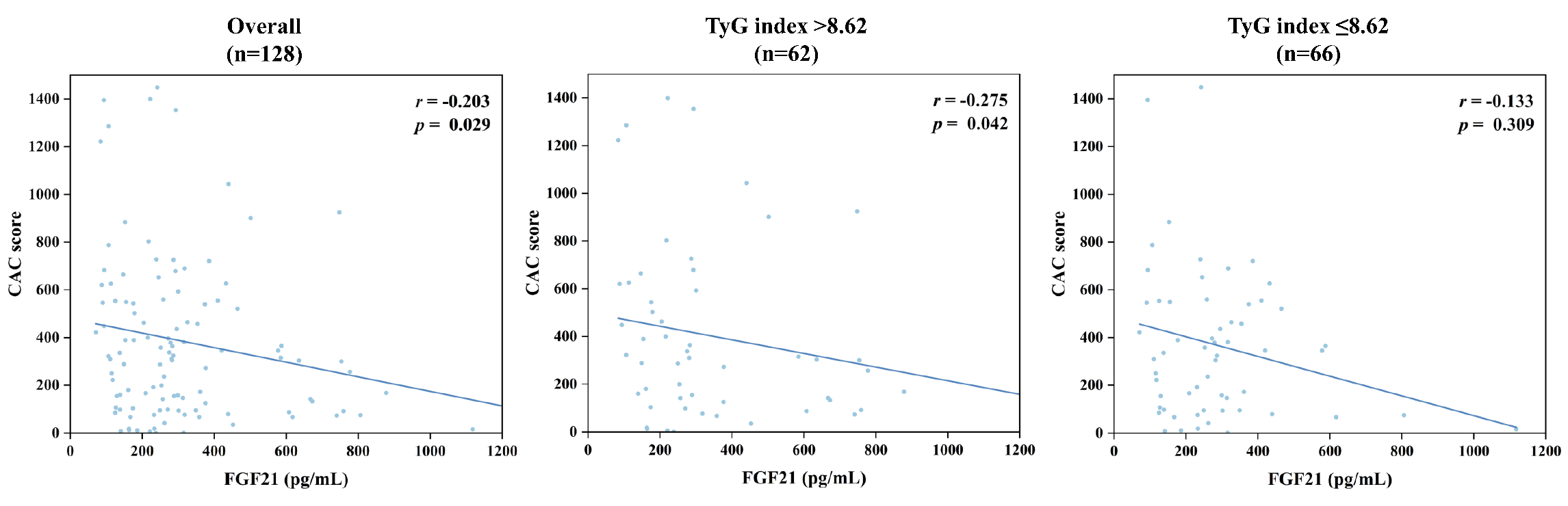


Supplementary Fig. 3 ROC curve analyses of serum FGF21 for diagnosing severe CAC in CAD patients with varying degrees of IR

Abbreviations: AUC, area under the curve; CAC, coronary artery calcification; CAD, coronary artery disease; FGF21, fibroblast growth factor 21; IR, insulin resistance; ROC, receiver operating characteristic. TyG, triglyceride-glucose index.


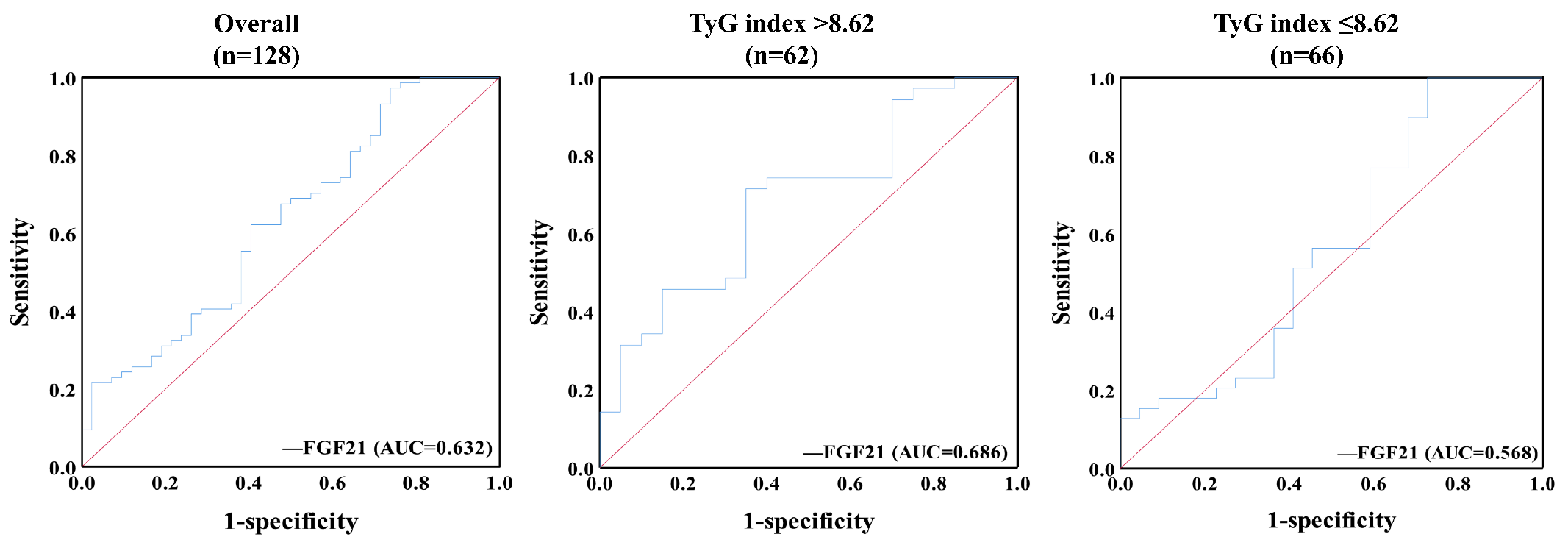


Supplementary Fig. 4 The restricted cubic spline curves for the association between FGF21 and severe CAC. (A) Overall; (B) TyG index >8.62; (C) TyG index ≤8.62

Abbreviations: CAC, coronary artery calcification; CI, confidence interval; FGF21, fibroblast growth factor 21.


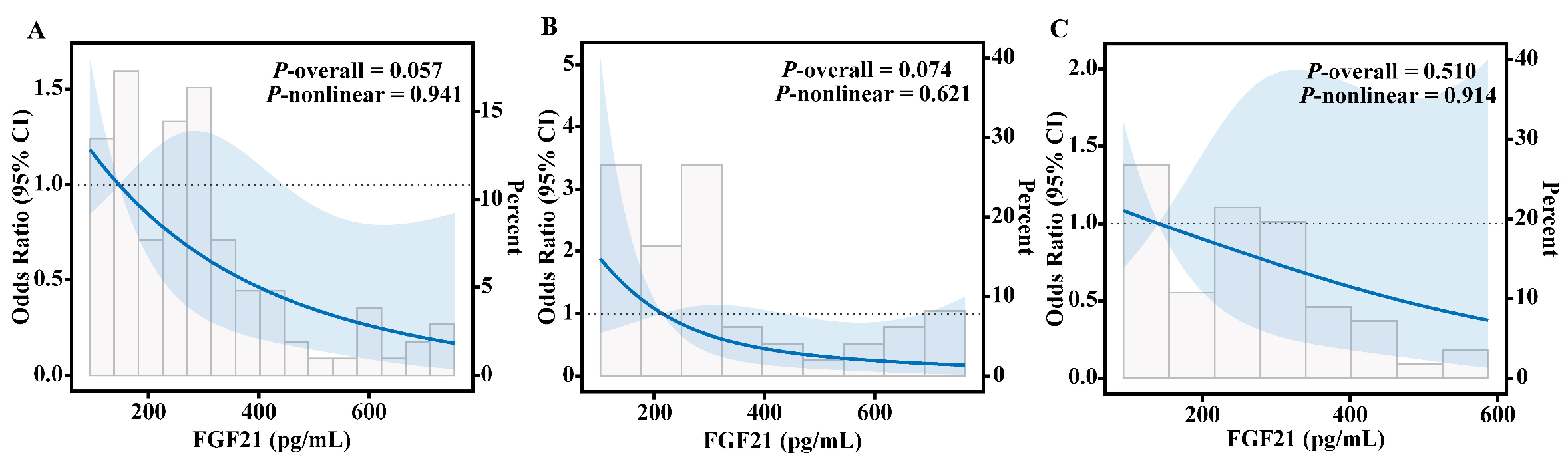

Supplement: Supplementary file 1 [file 2153-8174-27-3-46781-s1.zip › Supplementary Material.docx]
